# Codon Usage is Influenced by Compositional Constraints in Genes Associated with Dementia

**DOI:** 10.3389/fgene.2022.884348

**Published:** 2022-08-09

**Authors:** Taha Alqahtani, Rekha Khandia, Nidhi Puranik, Ali M. Alqahtani, Yahia Alghazwani, Saad Ali Alshehri, Kumarappan Chidambaram, Mohammad Amjad Kamal

**Affiliations:** ^1^ Department of Pharmacology, College of Pharmacy, King Khalid University, Abha, Saudi Arabia; ^2^ Department of Biochemistry and Genetics, Barkatullah University, Bhopal, India; ^3^ Department of Pharmacognosy, College of Pharmacy, King Khalid University, Abha, Saudi Arabia; ^4^ Institutes for Systems Genetics, Frontiers Science Center for Disease-related Molecular Network, West China Hospital, Sichuan University, Chengdu, China; ^5^ King Fahd Medical Research Center, King Abdulaziz University, Jeddah, Saudi Arabia; ^6^ Department of Pharmacy, Faculty of Allied Health Sciences, Daffodil International University, Dhaka, Bangladesh; ^7^ Enzymoics, Novel Global Community Educational Foundation, Hebersham, NSW, Australia

**Keywords:** dementia, GC composition, compositional constraint, codon usage, nucleotide skew

## Abstract

Dementia is a clinical syndrome characterized by progressive cognitive decline, and the symptoms could be gradual, persistent, and progressive. In the present study, we investigated 47 genes that have been linked to dementia. Compositional, selectional, and mutational forces were seen to be involved. Nucleotide components that influenced A- and GC-affected codon usages bias at all three codon positions. The influence of these two compositional constraints on codon usage bias (CUB) was positive for nucleotide A and negative for GC. Nucleotide A also experienced the highest mutational force, and GC-ending codons were preferred over AT-ending codons. A high bias toward GC-ending codons enhances the gene expression level, evidenced by the positive association between CAI- and GC-ending codons. Unusual behavior of the TTG codon showing an inverse relationship with the GC-ending codon and negative influence of gene expression, behavior contrary to all other GC-ending codons, shows an operative selectional force. Furthermore, parity analysis, higher translational selection value, preference of GC-ending codons over AT-ending codons, and association of gene length with gene expression refer to the dominant role of selection pressure with compositional constraint and mutational force-shaping codon usage.

## 1 Introduction

Dementia, a collection of illnesses characterized by a loss in cognitive ability that affects activities of daily living and social functioning, is one of the most severe worldwide health and social care concerns of the twenty-first century. Dementia affects approximately 50 million people globally, and the number is anticipated to rise by 2050, with one new case occurring every 3 s. Because of the rising number of people living with dementia, its significant social and economic impact, and the lack of a solution, countries must endeavor to reduce modifiable dementia risk factors (Dementia, 2022). Dementia is defined as losing two or more cognitive abilities that produce functional impairment but not alertness or attention. The deterioration in cognition distinguishes it from lifelong intellectual disability and learning problems, both present from birth and manifest in infancy. Dementia is a syndrome characterized by various brain diseases that cause memory, language, understanding, and judgment impairments (Kindell et al., 2017). Alzheimer’s disease (AD), vascular dementia, dementia with Lewy bodies, and frontotemporal dementia are the most frequent kinds of dementia (Hinz and Geschwind, 2017; Oh and Rabins, 2019). Despite breakthroughs in our understanding of the etiology, neuropathophysiology, and treatment of diverse types of dementia, these disorders continue to be significant and growing health concerns globally. Dementia affects many Parkinson’s disease patients, with a point prevalence of roughly 30%. Significant deficits characterize the executive, visuospatial, attention, and memory (Hanagasi et al., 2017). In dementia, neuropsychiatric symptoms are practically universal. The vast majority of persons with dementia will have at least one neuropsychiatric symptom throughout their illness (Radue et al., 2019). Interventions and care can dramatically improve the quality of life for people with dementia, their families, and society. There is a tangible link between cardiovascular disease, especially hypertension, and dementia (Cheng, 2017). A new study conducted in three French cities discovered a link between seven vascular risk factors and the probability of dementia (Hachinski, 2019). People with dementia are pre-dominantly susceptible to COVID-19 because of their oldness, multi-morbidity, and difficulties in keeping physical separation (Livingston et al., 2020).

In recent decades, tremendous progress has been made toward understanding the molecular genetics of neurodegenerative dementias and identifying the pathologically aggregating proteins implicated. It became possible mainly due to advances in sequencing technology and bioinformatics approaches (Hinz and Geschwind, 2017). Defects in specific genes that form abnormal proteins lead to unusual brain changes that cause neurodegenerative diseases.

Genome-wide association studies (GWAS) indicated the APOE as a decisive genetic risk factor (Harold et al., 2009; Lambert et al., 2009; Seshadri et al., 2010). UBQLN2 is another gene that encodes for ubiquitin-like protein ubiquitin two and is responsible for dominantly inherited, chromosome-X-linked amyotrophic lateral sclerosis (ALS). ALS, a paralytic disorder, results from motor-neuron degeneration in the brain and spinal cord (Deng et al., 2011). Frontotemporal lobar degeneration (FLD) is a degenerative disorder that is a genetically and pathologically heterogeneous neurodegenerative disorder. After AD, it is the most common cause of neurodegeneration, and a higher proportion of this disease is genetic causes. Mutations are present in six unrelated genes that are directly involved in FLD. Out of six, three more frequent genes are the tau gene MAPT, the progranulin gene GRN, and the hexanucleotide repeat expansions C9ORF72, TADRP, VCP, and CHMP2B are the other four genes (Paulson and Igo, 2011). Other GWAS indicated CLU gene encoding for clusterin, a protein involved in modulating the inflammatory response, as a potential risk factor, and its level is found elevated in AD patients (Lambert et al., 2009). ApoE, CLU, CR1, CD33, ABCA7, and MS4A are considered genes responsible for the late onset of AD genes (Bates et al., 2009). A large study conducted in South China encompassing 1795 patients with neurodegenerative dementias and pathogenic variants of PSEN1, PSEN2, APP, MAPT, GRN, CHCHD10, TBK1, VCP, HTRA1, OPTN, SQSTM1, and SIGMAR1 genes and abnormal repeat expansions in C9orf72 and HTT was observed, and among all, PSEN1 gene was mutated frequently (Jiao et al., 2021). The mutated genes result in the production of abnormal proteins. Amino acids are building blocks for proteins. Each amino acid is represented by a codon, a three-base sequence of nitrogenous bases. Synonymous variants with either decreasing or increasing RSCU scores in two (MLST8 and RHOB) and six genes (FLG2, CHD6, CD244, FLG-AS1, SERPINB5, and GTF3C1), respectively, have been found associated with entorhinal cortical thickness. In addition, also rare synonymous variants of MLST8 and RHOB genes were associated with the whole-brain cortical thickness (Miller et al., 2018).

Except for methionine and tryptophan, all amino acids are encoded by two or more than two codons, referred to as synonymous codons. However, synonymous codons are not used equal, with the preference of a few codons over others and termed codon usage bias (CUB). CUB has been discovered as a species-specific phenomenon. In mammals, the neutral concept and the selection–mutation–drift balance model are the two major theories to explain the origin of CUB. However, after the entire genome sequencing of numerous organisms, these two ideas were insufficient to explain the CUB phenomena. Other factors influencing CUB include the GC content (Newman et al., 2016), gene length (Duret and Mouchiroud, 1999), RNA and protein structures (Zhou et al., 2016), physical properties of encoded protein (Chen et al., 2017), environmental stress (Arella et al., 2021), tRNA population (Sabi and Tuller, 2014), etc. This research would reveal the molecular details of imperative genes participating in the regular operation of the central nervous system. In CUB, the GC content plays a crucial role in the bending, thermostability, and converting ability of B DNA to Z DNA. Newman et al. (2016) (Newman et al., 2016) found that after optimizing the codons, the improved rate of protein expression is not attributed to the enhanced translation rate but to improved transcription. An enhancement in transcription is owing to the enhanced guanine–cytosine (GC) content following codon optimization (Uddin and Chakraborty, 2019).

Furthermore, research suggests that synonymous variations can affect gene regulation pathways, particularly those connected to Alzheimer’s. Codon bias occurs when some synonymous codons are chosen over others. When it comes to codons utilized more or less frequently in the genome, bias may occur. Optimal and non-optimal codons, which have more significant and weaker codon and anti-codon interactions, can also cause a bias (Miller et al., 2018).

In the present study, we investigated the effects of various factors, including nucleotide composition, expression patterns, physical properties of the protein, length, and compositional constraints at various codon positions, CAI, length, and role of selectional and mutational pressure on the codon usage of 47 genes related to dementia. The genes envisaged here had a direct or indirect effect on neuronal health in case of improper functioning. Table 1 describes the occurrence of diseases if these genes are malfunctioning. The set of genes envisaged is involved in at least 42 diseased conditions, including but not limited to amyotrophic lateral sclerosis, Alzheimer’s disease, neurodegeneration with brain iron accumulation, Parkinson’s disease, frontotemporal lobar degeneration, cerebral amyloid angiopathy, lateral meningocele syndrome, Lewy body dementia, etc. The study will help determine various forces that drive the codon usage in genes involved in dementia.

**TABLE 1:** Gene associated with dementia.

S.No	Gene	Accession number	Synonyms	Location on chromosome	Disease associated with malfunctional gene	Function/s
1	ALS2 (Alsin Rho guanine nucleotide exchange factor)	NG_008775.1	ALSJ; PLSJ; IAHSP; ALS2CR6	2q33.1	Juvenile Amyotrophic Lateral Sclerosis	Functions as a guanine nucleotide exchange factor for the small GTPase RAB5
2	ANG (angiogenin)	NG_008717.2	ALS9, HEL168, RAA1, RNASE4, RNASE5	14q11.2	Amyotrophic Lateral Sclerosis 1	A potent mediator of new blood vessel formation
3	APP (amyloid beta precursor protein)	NG_007376.2	AAA, ABETA, ABPP, AD1, APPI, CTF gamma, CVAP, PN-II, PN2, alpha-sAPP, preA4	21q21.3	Alzheimer Disease	Have bactericidal and antifungal activities
4	C19orf12 (chromosome 19 open reading frame 12)	NG_031970.2	MPAN; NBIA3; NBIA4; SPG43	19q12	Neurodegeneration with Brain Iron Accumulation	Mutations in this gene are a cause of neurodegeneration with brain iron accumulation-4 (NBIA4)
5	C9orf72 (C9orf72-SMCR8 complex subunit)	NG_031977_2	ALSFTD, DENND9, DENNL72, FTDALS, FTDALS1	9p21.2	Amyotrophic Lateral Sclerosis 1	Plays an important role in the regulation of endosomal trafficking
6	CHCHD10 (coiled-coil-helix-coiled-coil-helix domain containing 10)	NG_034223_1	IMMD; SMAJ; MIX17A; FTDALS2; N27C7-4; C22orf16	22q11.23	Frontotemporal Dementia and/or Amyotrophic Lateral Sclerosis 2	Role in cristae morphology maintenance or oxidative phosphorylation
7	CHMP2B (charged multivesicular body protein 2B)	NG_007885_1	DMT1; ALS17; VPS2B; VPS2-2; CHMP2.5; FTDALS7	3p11.2	Amyotrophic Lateral Sclerosis 1	Functions in the recycling or degradation of cell surface receptors
8	CLCN3 (chloride voltage-gated channel 3)	NG_029731_1	CLC3; ClC-3	4q33	Cystic Fibrosis	Essential for lysophosphatidic acid (LPA)-activated Cl- current activity and fibroblast-to-myofibroblast differentiation
9	CLCN5 (chloride voltage-gated channel 5)	NG_007159_3	XRN; CLC5; XLRH; CLCK2; ClC-5; DENT1; DENTS; NPHL1; NPHL2; hCIC-K2	Xp11.23	Hypophosphatemic Rickets, X-Linked Recessive	Facilitate albumin uptake by the renal proximal tubule
10	CP (ceruloplasmin)	NG_011800_3	CP-2	3q24-q25.1	Aceruloplasminemia	It binds most of the copper in plasma and is involved in the peroxidation of Fe(II) transferrin to Fe(III) transferrin
11	CTSD (cathepsin D)	NG_012303_2	CPSD; CLN10; HEL-S-130P	11p15.5	Alzheimer Disease	It exhibits pepsin-like activity and plays a role in protein turnover and in the proteolytic activation of hormones and growth factors
12	CTSF (cathepsin F)	NG_008655_1	CATSF; CLN13	11q13.2	Adult Neuronal Ceroid Lipofuscinosis	Targeted to the endosomal/lysosomal compartment via the mannose 6-phosphate receptor pathway
13	EIF4G1 (eukaryotic translation initiation factor 4 gamma 1)	NG_032973_1	P220; EIF4F; EIF4G; EIF4GI; PARK18; EIF-4G1	3q27.1	Parkinson Disease 18, Autosomal Dominant	Facilitates the recruitment of mRNA to the ribosome
14	ERBB4 (erb-b2 receptor tyrosine kinase 4)	NG_016850_1	HER4; ALS19; p180erbB4	2q34	Amyotrophic Lateral Sclerosis 19	Binds to and is activated by neuregulins and other factors and induces a variety of cellular responses including mitogenesis and differentiation
15	FUS (FUS RNA binding protein)	NG_011805_2	ALS6, ETM4, FUS1, HNRNPP2, POMP75, TLS, altFUS	16p11.2	Frontotemporal Dementia and/or Amyotrophic Lateral Sclerosis 1	This protein belongs to the FET family of RNA-binding proteins, which have been implicated in cellular processes that include the regulation of gene expression, maintenance of genomic integrity, and mRNA/microRNA processing
16	GRN (granulin precursor)	NG_012889_2	GEP; GP88; PEPI; PGRN; CLN11; PCDGF	17q21.31	Grn-Related Frontotemporal Lobar Degeneration	It regulates cell growth
17	HNRNPA1 (heterogeneous nuclear ribonucleoprotein A1)	NG_007886_1	ALS19, ALS20, HNRPA1, HNRPA1L3, IBMPFD3, UP 1, hnRNP A1, hnRNP-A1	12q13.13	Amyotrophic Lateral Sclerosis 1	Plays a key role in the regulation of alternative splicing
18	ITM2B (integral membrane protein 2B)	NG_033830_1	BRI; FBD; ABRI; BRI2; E25B; E3-16; RDGCA; imBRI2; BRICD2B	13q14.2	Cerebral Amyloid Angiopathy, Itm2b-Related, 2	Inhibits the deposition of beta-amyloid
19	MAPT (microtubule-associated protein tau)	NG_013069_2	DDPAC, FTDP-17, MAPTL, MSTD, MTBT1, MTBT2, PPND, PPP1R103, TAU, tau-40	17q21.31	Parkinson–Dementia Syndrome	Regulates alternative splicing, giving rise to several mRNA species
20	NOTCH3 (notch receptor 3)	NG_007398_2	IMF2; LMNS; CASIL; CADASIL; CADASIL1	19p13.12	Lateral Meningocele Syndrome	Plays a key role in neural development
21	NPC1 (NPC intracellular cholesterol transporter 1)	NG_009819_1	NPC, POGZ, SLC65A1	18q11.2	Niemann–Pick Disease, Type C1	Transports low-density lipoproteins to late endosomal/lysosomal compartments
22	NPC2 (NPC intracellular cholesterol transporter 2)	NG_012795_1	HE1; EDDM1	14q24.3	Niemann–Pick Disease, Type C2	The encoded protein may function in regulating the transport of cholesterol through the late endosomal/lysosomal system
23	CSF1R (colony-stimulating factor 1 receptor)	NG_007117_1	BANDDOS, C-FMS, CD115, CSF-1R, CSFR, FIM2, FMS, HDLS, M-CSF-R	5q32	Leukoencephalopathy	Controls the production, differentiation, and function of macrophages
24	OPTN (optineurin)	NG_012876_1	ALS12, FIP2, GLC1E, HIP7, HYPL, NRP, TFIIIA-INTP	10p13	Amyotrophic Lateral Sclerosis	Functions in cellular morphogenesis and membrane trafficking, vesicle trafficking, and transcription activation
25	PDGFB (platelet-derived growth factor subunit B)	NG_012111_1	SIS; SSV; IBGC5; PDGF2; c-sis; PDGF-2	22q13.1	Dermatofibrosarcoma Protuberans	These proteins bind and activate PDGF receptor tyrosine kinases, which play a role in a wide range of developmental processes
26	PDGFRB (platelet-derived growth factor receptor beta)	NG_023367_1	IMF1; KOGS; IBGC4; JTK12; PDGFR; PENTT; CD140B; PDGFR1; PDGFR-1	5q32	Infantile myofibromatosis	This gene is essential for normal development of the cardiovascular system and aids in rearrangement of the actin cytoskeleton
27	PPT1 (palmitoyl-protein thioesterase 1)	NG_009192_1	PPT; CLN1; INCL	1p34.2	Neuronal Ceroid Lipofuscinosis	Enzyme removes thioester-linked fatty acyl groups such as palmitate from cysteine residues
28	PRKAR1B (protein kinase cAMP-dependent type I regulatory subunit beta)	NG_042811_1	PRKAR1	7p22.3	Prkar1b-Related Neurodegenerative Dementia with Intermediate Filaments	Involved in many cellular events including ion transport, metabolism, and transcription
29	PRNP (prion protein)	NG_009087_1	ASCR, AltPrP, CD230, CJD, GSS, KURU, PRIP, PrP, PrP27-30, PrP33-35C, PrPc, p27-30	20p13	Huntington disease-like 1	Mutations in the repeat region as well as elsewhere in this gene have been associated with the Creutzfeldt–Jakob disease, fatal familial insomnia, Gerstmann–Straussler disease, and kuru
30	PSEN1 (presenilin 1)	NG_007386_2	AD3; FAD; PS1; PS-1; S182; ACNINV3	14q24.2	Alzheimer Disease 3	Presenilins are involved in the cleavage of the Notch receptor
31	PSEN2 (presenilin 2)	NG_007381_2	AD4; PS2; AD3L; STM2; CMD1V	1q42.13	Alzheimer Disease 4	This is involved in the cleavage of the Notch receptor in a way that they either directly regulate gamma-secretase activity or themselves act as protease enzymes
32	SERPINI1 (serpin family I member 1)	NG_008217_1	PI12; HNS-S1; HNS-S2; neuroserpin	3q26.1	Dementia, Alzheimer Disease	Plays a role in the regulation of axonal growth and the development of synaptic plasticity
33	SETX (senataxin)	NG_007946_1	ALS4; AOA2; Sen1; SCAN2; SCAR1; bA479K20.2	9q34.13	Amyotrophic Lateral Sclerosis 4, Juvenile	Involved in both DNA and RNA processing
34	SIGMAR1 (sigma non-opioid intracellular receptor 1)	NG_029945_2	SRBP; ALS16; DSMA2; OPRS1; SR-BP; SIG-1R; SR-BP1; sigma1R; hSigmaR1	9p13.3	Juvenile Amyotrophic Lateral Sclerosis	Plays an important role in the cellular functions of various tissues associated with the endocrine, immune, and nervous systems
35	SNCA (synuclein alpha)	NG_011851_1	NACP, PARK1, PARK4, PD1	4q22.1	Parkinson Disease 1, Autosomal Dominant	Inhibits phospholipase D2 selectively; serves to integrate presynaptic signaling and membrane trafficking
36	SNCB (synuclein beta)	NG_012131_1	Synuclein beta	5q35.2	Dementia, Lewy Body	It inhibits phospholipase D2 and may function in neuronal plasticity
37	SOD1 (superoxide dismutase 1)	NG_008689_1	ALS, ALS1, HEL-S-44, IPOA, SOD, STAHP, hSod1, homodimer	21q22.11	Amyotrophic Lateral Sclerosis 1	SOD1 contains an antimicrobial peptide that displays antibacterial, antifungal, and anti-MRSA activities
38	SORL1 (sortilin related receptor 1)	NG_023313_1	C11orf32, LR11, LRP9, SORLA, SorLA-1, gp250	11q24.1	Alzheimer Disease	Plays roles in endocytosis and sorting
39	SPG11 (SPG11 vesicle trafficking associated, spatacsin)	NG_007117_1	ALS5; CMT2X; KIAA1840	15q21.1	Juvenile Amyotrophic Lateral Sclerosis	Potential transmembrane protein that is phosphorylated upon DNA damage
40	SQSTM1 (sequestosome 1)	NG_011342_1	p60; p62; A170; DMRV; OSIL; PDB3; ZIP3; p62B; NADGP; FTDALS3	5q35.3	Sporadic and familial Paget disease of bone	Protein functions as a scaffolding/adaptor protein i
41	TARDBP (TAR DNA binding protein)	NG_008734_1	ALS10, TDP-43	1p36.22	Amyotrophic Lateral Sclerosis 10 with or Without Frontotemporal Dementia	Regulates alternate splicing of the CFTR gene
42	TREM2 (triggering receptor expressed on myeloid cells 2)	NG_011561_1	PLOSL2; TREM-2; Trem2a; Trem2b; Trem2c	6p21.1	Alzheimer Disease	Functions in immune response and may be involved in chronic inflammation by triggering the production of constitutive inflammatory cytokines
43	TYROBP (transmembrane immune signaling adaptor TYROBP)	NG_009304_1	DAP12; KARAP; PLOSL; PLOSL1	19q13.12	Dementia	It associates with the killer-cell inhibitory receptor (KIR) family of membrane glycoproteins and may act as an activating signal transduction element
44	UBQLN2 (ubiquilin 2)	NG_016249_1	DSK2; ALS15; CHAP1; N4BP4; PLIC2; HRIHFB2157	Xp11.21	Amyotrophic Lateral Sclerosis 1	Functionally links the ubiquitination machinery to the proteasome to affect *in vivo* protein degradation
45	VCP (valosin containing protein)	NG_007887_1	CDC48, FTDALS6, TERA, p97	9p13.3	Amyotrophic Lateral Sclerosis 1	The encoded protein plays a role in protein degradation, intracellular membrane fusion, DNA repair and replication, regulation of the cell cycle, and activation of the NF-kappa B pathway
46	VPS13A (vacuolar protein sorting 13 homolog A)	NG_008931_1	CHAC, CHOREIN	9q21.2	Choreoacanthocytosis	It may control steps in the cycling of proteins through the trans-Golgi network to endosomes, lysosomes, and the plasma membrane
47	XPR1 (xenotropic and polytropic retrovirus receptor 1)	NG_050964_1	X3; SYG1; IBGC6; SLC53A1	1q25.3	Basal Ganglia Calcification, Idiopathic, 6	Involved in phosphate homeostasis by mediating phosphate export from the cell

## 2 Materials and Methods

### 2.1 Data Retrieval

Based on the information available at NCBI Genetic Testing Registry, 47 gene sequences involved in dementia (list of genes given in Table 1) were retrieved. Genetic testing is commercially available for all these genes from Amsterdam UMC Genome Diagnostics, Amsterdam University Medical Center, Netherlands, in conditions/phenotype of Alzheimer’s disease types 1–4, ABri amyloidosis, ADan amyloidosis, and amyotrophic lateral sclerosis. A total of 54 genes was available, and out of them, we took 47 gene sequences based on qualifying criteria. All the coding sequences were qualified based on nucleotides in triplicate, the absence of in-frame stop codons, and the lack of ambiguous nucleotides. Sequences less than 150 base pairs were also omitted.

### 2.2 Nucleotide Composition Analysis

The nucleotide composition analysis was done for genes related to dementia. Individually overall, %A, %T, %G, and %C nucleotide compositions were determined. Also, their compositions at all the three positions of codons were determined. Percent GC3 and the average composition of %GC at the first and second positions of codon (%GC12) were determined for neutrality analysis. Nucleotide composition at the third codon position was used for determining the AT bias [A3/(A3 + T3)] and GC bias [G3/(G3 + C3)] calculations to be included in parity analysis. The analysis was done using CAIcal, a web-based server available at http://genomes.urv.es/CAIcal (CAI calculator, 2022).

### 2.3 Dinucleotide Analysis

Sixteen dinucleotide combinations are possible with four nucleotides, but their appearance is biased in any genome. The ratio of the obtained-to-expected frequency is called the odds ratio. An odds ratio below 0.78 is called under-representation, and above 1.23, it is called over-representation of a dinucleotide (Butt et al., 2014).

### 2.4 RSCU Analysis

Among 64 codons (nucleotide triplets) in the standard genetic code, except for three stop codons (TAA, TAG, TGA), methionine, and tryptophan (Belalov and Lukashev, 2013), all amino acids are encoded by two or more than two triplets and are termed as synonymous codons. All the synonymous codons are not used equally, referred to as codon usage bias (CUB). The ratio observed to the expected frequency of a codon coding for an amino acid is termed as the relative synonymous codon usage (RSCU) value. RSCU analysis was done by CodonW 1.4.4 software available at http://codonw.sourceforge.net. The codons with RSCU values above 1.6 are called over-represented and below 0.6 are called under-represented (Kumar et al., 2021).

### 2.5 Codon Adaptation Index

Codon adaptation index measures the level of expression of a protein and the adaptiveness of a gene to the host. It is also a measure of CUB for a DNA/RNA sequence and can quantify the codon usage similarities between a gene and a reference set (Puigbò et al., 2008). Its values range between 0 and 1. If a gene always uses the most frequently used synonymous codon from the reference set, in such case, the CAI value will be 1. In contrast, the usage of the least frequently used synonymous codon from the reference set will result in a CAI value of zero. CAI values were obtained by the software developed by Bourret et al. (2019) (Bourret et al., 2019). As a reference set, the RSCU analysis of 40662582 codons belonging to 93487 coding sequences from Homo sapiens available at the codon usage database https://www.kazusa.or.jp/codon/ (Codon Usage Database, 2022) was used.

### 2.6 Scaled Chi-Square

It is a directional estimate of CUB and computed as the sum of the chi-square values of the codon families within the gene normalized by peptide length termed as scaled chi-square (). For each gene, SCS was calculated. Its value ranges between 0 and 1, and a higher value shows higher bias (Chu and Wei, 2019).

### 2.7 Protein Indices Calculation

The physical properties of proteins affect several of the properties of the protein and its biological functions. To determine the relationship of the physical properties of proteins with CUB, a correlation analysis was carried out between various protein indices (PIs or isoelectric point, instability index, aliphatic index, and hydrophobicity, frequency of acidic, basic, neutral amino acids, GRAVY and AROMA; total nine) and SCS. The protein properties were calculated using Protparam Expasy (Gasteiger et al., 2005) and peptide2 tool available at Peptide 2.0 Inc. GRAVY and AROMA values were calculated using the software developed by Bourret et al. (2019) (Bourret et al., 2019). GRAVY expresses features of both hydrophobicity and hydrophilicity, and it ranges between -2 and +2. A positive value is an indicator of a more hydrophobic protein and vice versa. AROMA indicates the frequency of aromatic amino acids. Hydrophobicity measures a protein’s solubility and plays a role in the protein–protein interactions. The aliphatic index is a suggestive of volume gained by aliphatic side chains. An instability index with an aliphatic index reveals the stability of a protein (Khandia et al., 2021). The isoelectric point is a value where no net electric charge on protein is present, and solubility is minimal (ScienceDirect Topics, 2022).

### 2.8 Calculation of Skews

AT skew, GC skew, purine skew, pyrimidine skew, amino skew, and keto skew are determinants of compositional skews and are calculated using the formula proposed by Wu et al. (2021). Cumulative GC and AT skews are calculated as a sum of (G-C)/(G + C) and (A-T)/(A + T), respectively (Grigoriev, 1998). Likewise, keto-amino or purine-pyrimidine skews were obtained by making appropriate replacements (Powdel et al., 2010).

### 2.9 Neutrality plot

To generate a regression plot, the mean %GC12 and %GC3 were plotted on the *Y-* and *X*-axes. The neutrality plot measures the mutational force or the neutrality primarily. When the slope is 1, codon usage is solely driven by mutational forces (Guan et al., 2018). Conversely, a slope deviation from 1 indicates other forces like selection pressure while shaping codon usage in any organism.

### 2.10 Parity Plot

A parity rule 2 (PR2) bias was calculated to determine the disparity of the usage of AT or GC at the third position of the codon. A plot is made by taking the average AT bias [A3/(A3 + T3)] as the ordinate and GC bias [G3/(G3 + C3)] as the abscissa (K handia et al., 2019), and a scatter plot is made. At the center of the plot, where the value is zero, A = T and C = G in a strand (Yang et al., 2015).

### 2.11 Effect of the Mutation on Compositional Parameters

A plot between the overall nucleotide content and nucleotide content at the third codon position was plotted for all the four nucleotides (%A3-%A, %T3-%T, %C3-%C, %G3-%G). It is indicative of the effect of mutational force on the composition of the gene.

### 2.12 Translational Selection

Translational selection (P2) is a measure of the codon–anticodon association and the translational efficacy of a gene (Bennetzens and Hall, 1982). Translational selection P2 was calculated using the formula

$$\text{P}2=\frac{\text{WWC}+\text{SSU}}{\text{WWY}+\text{SSY}}$$

where W = A or U, S = C or G, and Y = C or U.

And the values above 0.5 are indicative of a bias favoring translational selection (A U et al., 2019).

## 3 Results

### 3.1 Compositional Analysis

**FIGURE 1:**
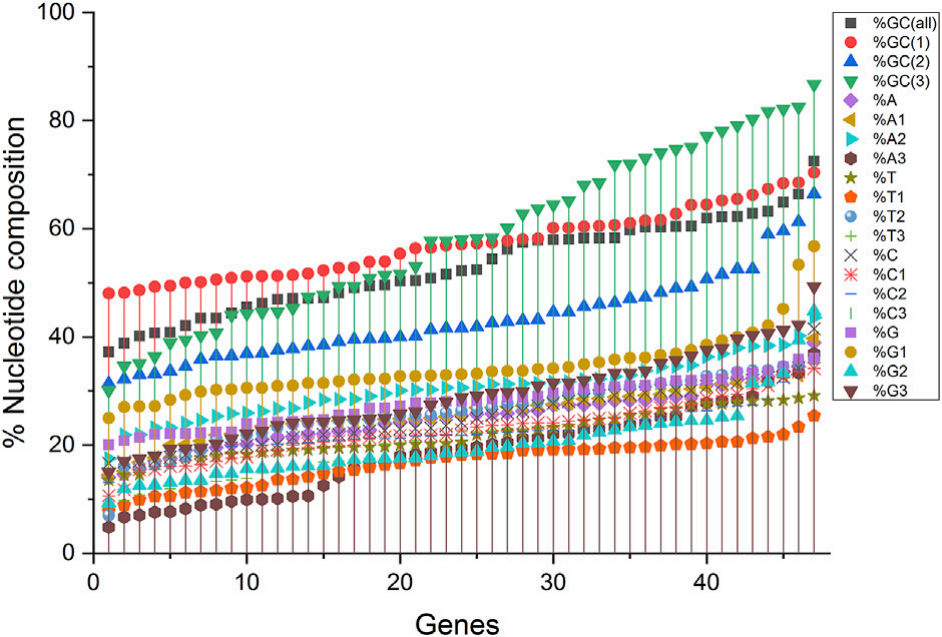
A nucleotide compositional analysis figure shows the trend of the compositional constraints. The nucleotide contents are sorted here as per their increasing values and do not depict their content in a single gene.

Compositional analysis revealed that among all nucleotide compositions, the %GC3 content was highly variable, ranging between 30.08 and 86.68%. Overall, the average percentage composition analysis revealed that %GC1 and %GC3 compositions were almost equal (57.17 and 58.51%, respectively), while the %GC2 composition was the least (43.21%). The average nucleotide composition for %A and %C nucleotides was almost equal (25.09 and 25.12%, respectively), with the maximum for %C (27.84%) and least for %T (21.94). %T1 was the least at codon position one (T composition at codon position one and likewise), and %A3 had a trend of low appearance in composition (Figure 1).

### 3.2 Relationship of CUB and Protein Indices

The SCS correlated only with the frequency of acidic amino acids. It had a positive association with the frequency of acidic amino acids (r = 0.373, *p* < 0.001). The correlation was absent for any of the other eight tested indices in the present study.

### 3.3 Compositional Disproportion Affects CUB

The effect of compositional disproportion on CUB was observed by correlating the AT skew, GC skew, purine skew, pyrimidine skew, amino skew, and keto skew with SCS. A significantly strong positive relationship between CUB and purine skew (r = 0.535, *p* < 0.001), pyrimidine skew (r = 0.407,*p* < 0.01), amino skew (r = 0.370, *p* < 0.05), and keto skew (r = 0.535,*p* < 0.001) was found.

### 3.4 Compositional Constraint affects CUB

Correlation analysis was carried out between the nucleotide content and SCS values (Table 2). Nucleotide A has a positive correlation with CUB at all three positions of codons. Conversely, %GC composition had a negative association with CUB at all codon positions. At other codon positions, also a few correlations were observed. Overall analysis revealed that compositional constraints affect CUB.

**TABLE 2:** Correlation between nucleotide compositions and CUB.

	%A	%A1	%A2	%A3	%T	%T1	%T2
Pearson (r)	0.411	0.374	0.450	0.299	0.460	0.384	0.052
*p* value	**	**	**	*	**	**	NS
	%T3	%C	%C1	%C2	%C3	%G	%G1
Pearson (r)	0.479	−0.306	−0.266	−0.288	−0.233	−0.581	−0.332
*p* value	***	*	NS	*	NS	***	*
	%G2	%G3	%GC	%GC1	%GC2	%GC3	%GC3s
Pearson (r)	−0.188	−0.531	−0.501	−0.548	−0.342	−0.414	−0.419
*p* value	NS	***	***	***	*	**	**

***p< 0.001. **p < 0.01, *p < 0.05, NS, Non-significant.

### 3.5 Effects of Compositional Constraints on Protein Indices

Out of nine protein indices studied, %GC2 had a significant relationship with six indices (both positive and negative) while %G2 and %T2 had a relationship (both positive and negative) with five indices each. %GC2 had a positive association with PI (r = 0.356, *p* < 0.05), instability index (r = 0.413, *p* < 0.01), and frequency of neutral amino acid (r = 0.686, *p* < 0.05), while a negative association with aliphatic index (r = -0.644, *p* < 0.001), hydrophobicity (r = -0.361, *p* < 0.05), and frequency of acidic amino acid (r = −0.554, *p* < 0.001). Alike GC2, %G2 also had a negative association with aliphatic index (r = −.656, *p* < 0.001), hydrophobicity (r = −0.646, *p* < 0.001), and frequency of acidic amino acid (r = −0.501, *p* < 0.001), but a positive association with only PI (r = 0.497, *p* < 0.001) and frequency of neutral amino acid (r = 0.852, *p* < 0.001). %T2 had a positive relationship with aliphatic index (r = 0.938, *p* < 0.001) and hydrophobicity (r = 0.747, *p* < 0.001), while a negative relationship with PI (r = −0.548, *p* < 0.001), instability index (r = −0.427, *p* < 0.01), and frequency of neutral amino acid (r = −0.324, *p* < 0.05). %T1, %T3, %G3, %GC1, and %GC3 had no relationship with any of the protein indices tested in the present study. GRAVY has a positive association with %T1 (r = 0.340, *p* < 0.05) and %T2 (r = 0.801, *p* < 0.001), while a negative association with %A2 (r = −0.521, *p* < 0.001) and %G2 (r = −0.341, *p* < 0.05). AROMA has a positive association with nucleotide T at all the three positions of codon (%T1-r = 0.665, *p* < 0.001; %T2-r = 0.419, *p* < 0.01 and %T3- (r = 0.304, *p* < 0.05). AROMA had a negative association with %C2 (r = −0.498, *p* < 0.001), %G1 (r = −0.288, *p* < 0.05), %G3 (r = −0.304, *p* < 0.05), and %GC1 (r = −0.476, *p* < 0.01).

### 3.6 Dinucleotide Odds Ratio and its Impact on CUB

Analysis of the trend of dinucleotides in different genes associated with dementia showed that dinucleotides ApG, CpA, and TpG were either over or randomly represented (odds ratio >0.78). In contrast, CpG, GpT, and TpA dinucleotides were either under or randomly expressed based on the odds ratio (odds ratio <1.6). An analysis of the relationship between the dinucleotide odds ratio and CUB revealed that CUB has a significant positive association with ApA (r = 0.358, *p* < 0.05), ApC (r = 0.292, *p* < 0.05), ApT (r = 0.456, *p* < 0.01), TpA (r = 0.484, *p* < 0.001), and TpT (r = 0.466, *p* < 0.001) dinucleotides, while a negative relationship with CpG (r = −0.456, *p* < 0.001), GpC (r = −0.468, *p* < 0.001), and GpG (r = −0.621, *p* < 0.001) dinucleotides. There was no correlation between CpG and TpG dinucleotides (r = −0.091, *p* = 0.542).

### 3.7 RSCU Pattern Analysis Indicated Over-representation of GC-Ending Codons Over AT-Ending Codons

RSCU pattern analysis indicated the preference of GC-ending codons over AT-ending codons. Nucleotide CTG and GTG were over-represented in 74.46 and 68.08% of genes, respectively. GTA, CAA, CTA, ATA, TTA, CGT, GCG, ACG, CCG, and TCG codons were under-represented in 68.085, 59.57%, 72.34%, 70.216, 68.08, 53.19, 72.34, 57.44, 70.21, and 78.72% of genes, respectively. When RSCU values of 47 genes were correlated with SCS, SCS was found positively associated with a few AT-ending codons while negatively associated with some of the GC-ending codons (data not shown here).

### 3.8 RSCU Association with the Gene Expression Profile

**FIGURE 2:**
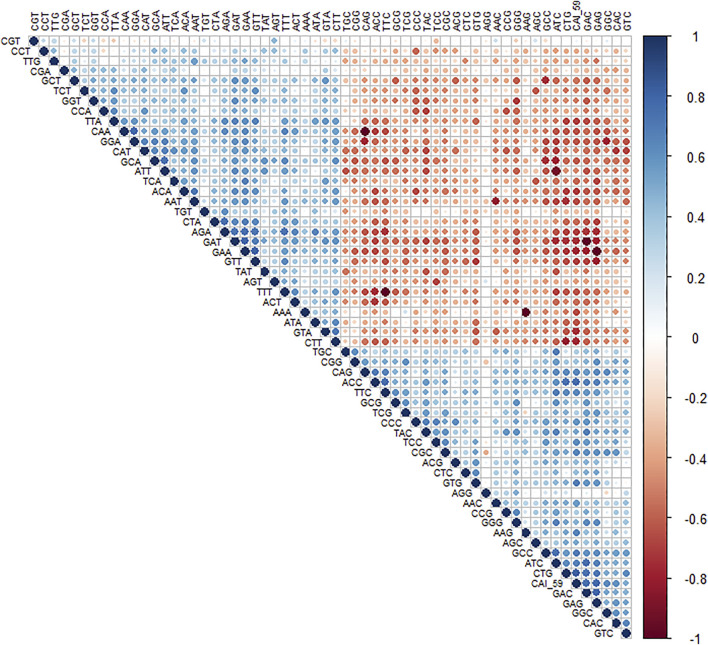
Correlation analysis of RSCU values of a codon and CAI value. A positive correlation is blue circles, while a negative correlation is depicted as red circles. The level of significance was at 0.05%.

To understand the trend of gene expression with AT- and CG-ending codons, the correlation analysis between RSCU values of genes and CAI (Figure 2) was performed. CAI value of genes is given in (Table 3).

**TABLE 3:** CAI values of the genes envisaged in the study.

S.No.	Gene	CAI		Gene	CAI		Gene	CAI
1	ALS2	0.719	17	GRN	0.828	33	SETX	0.705
2	ANG	0.784	18	HNRNPA1	0.771	34	SIGMAR1	0.788
3	APP	0.787	19	ITM2B	0.759	35	SNCA	0.762
4	C19orf12	0.796	20	MAPT	0.797	36	SNCB	0.803
5	C9orf72	0.686	21	NOTCH3	0.813	37	SOD1	0.774
6	CHCHD10	0.812	22	NPC1	0.756	38	SORL1	0.793
7	CHMP2B	0.694	23	NPC2	0.719	39	SPG11	0.721
8	CLCN3	0.709	24	OPTN	0.748	40	SQSTM1	0.8
9	CLCN5	0.732	25	PDGFB	0.819	41	TARDBP	0.74
10	CP	0.743	26	PDGFRB	0.816	42	TREM2	0.804
11	CSF1R	0.829	27	PPT1	0.74	43	TYROBP	0.759
12	CTSD	0.849	28	PRKAR1B	0.826	44	UBQLN2	0.744
13	CTSF	0.817	29	PRNP	0.798	45	VCP	0.77
14	EIF4G1	0.772	30	PSEN1	0.719	46	VPS13A	0.673
15	ERBB4	0.759	31	PSEN2	0.808	47	XPR1	0.742
16	FUS	0.782	32	SERPINI1	0.717	—	—	—

**FIGURE 3:**
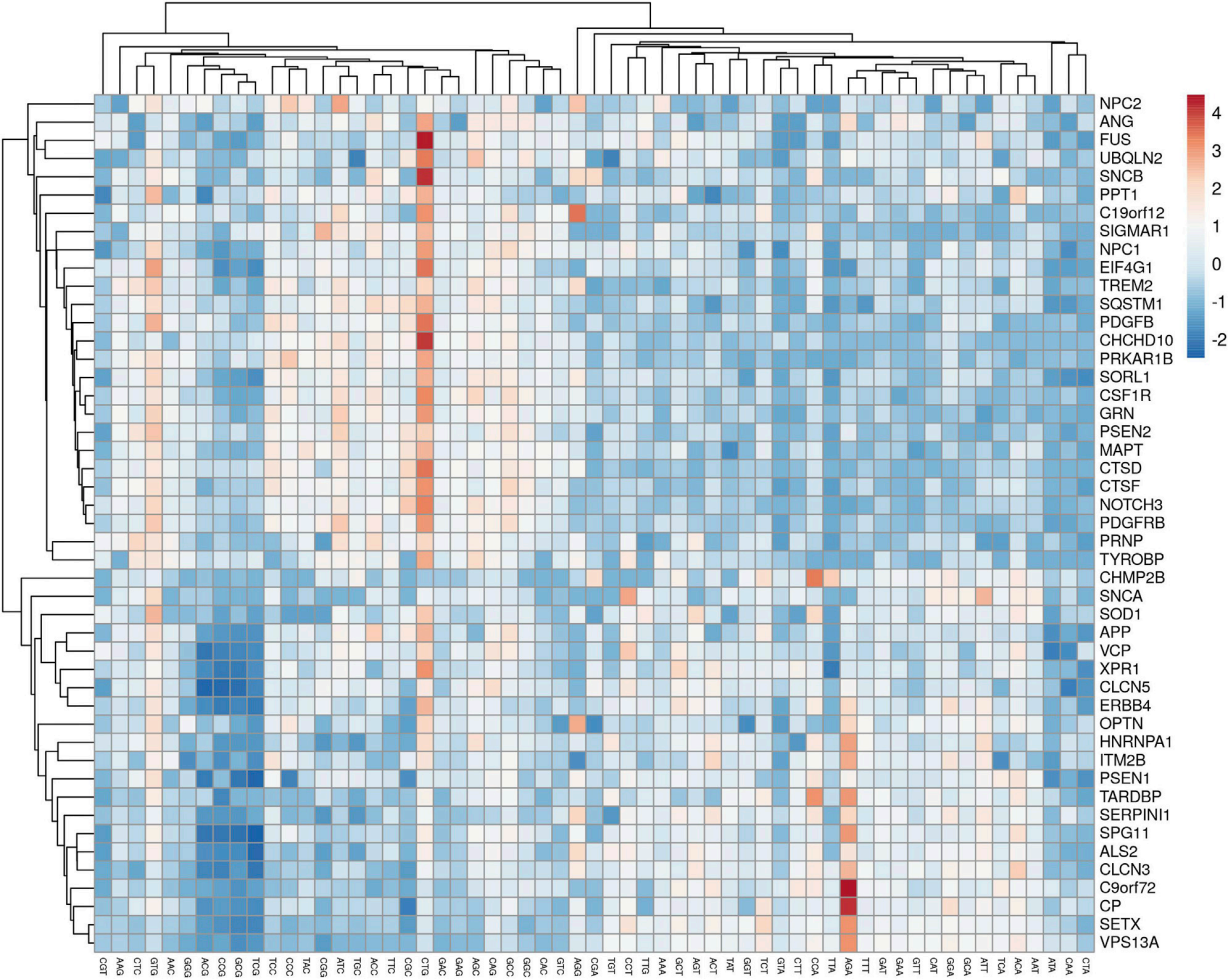
A cluster analyses of multivariate data showed the clustering of TTG with AT-ending codons.

The analysis revealed that gene expression was negatively correlated with all the AT-ending codons while positively correlated with GC-ending codons. Clustering of multivariate data based on the RSCU analysis revealed that codon TTG was clustered with GC-ending codons (Figure 3).

Codon TTG was inversely related to gene expression and surprisingly showed a negative association with GC-ending codons. Codons CGT and AGG had correlations only with two codons out of 59 (excluding trp, met, and stop codons). Codon CGT had a positive affiliation with AGT (r = 0.448, *p* < 0.05) and negative with TCC (r = −0.307, *p* < 0.05), while AGG codon had a positive affiliation with CCG (r = 0.303, *p* < 0.05) and negative with CGC (r = −0.358, *p* < 0.05). Here, the determination of codon correlation is important since codon correlation tends to accelerate the translation process compared to anti-correlated codons, and translation speed may be primarily explained based on codon correlation (Cannarozzi et al., 2010).

### 3.9 Unusual Behavior of CGT and AGG Codons Remains Unaffected by Compositional Constraints

**FIGURE 4:**
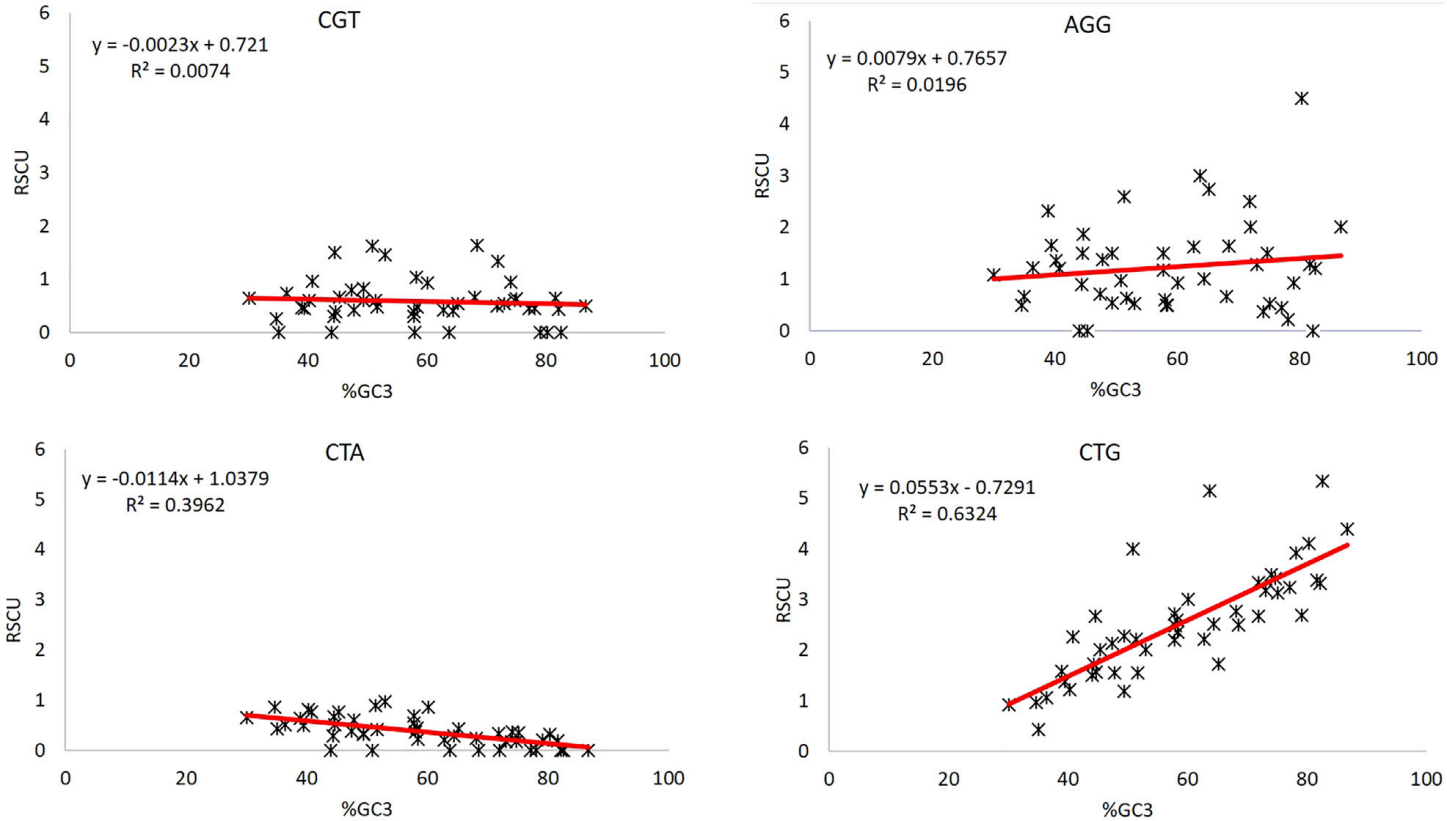
Regression analysis between GC3 and RSCU of codons CGT, AGG, CTA, and CTG.

GC composition can be used as both a good indicator of CUB (Shen et al., 2015a) and the extent of base composition (Das and Roy, 2021a). The unusual behavior of codons CGT and AGG can be further investigated for the impact posed by compositional bias. GC3 is a good indicator of compositional bias (Deka and Chakraborty, 2016); therefore, regression analysis between RSCU values of CGT and AGG, and %GC3 composition was done to evaluate the effect of compositional bias (Figure 4). We took two more codons, CTG and CTA, the most over-represented and under-represented codons. The R2 values here explain the percent variation in the RSCU value by the GC3 component. CTG and CTA, the most over-represented and under-represented codons, respectively, in the set of 47 genes, showed R2 values = 0.632 and 0.396, indicating that 63.2 and 39.6% variations in the CTG and CTA codon could be explained by %GC3 composition. Contrary to this, codons CGT and AGG could explain only 0.74 and 1.96% variations in %GC3, which is negligible. Furthermore, the regression analysis of these codons with AT3 revealed the same results. Hence, bias in these two codons CGT and AGG is not influenced by compositional constraints (Table 4).

**TABLE 4:** Effect of compositional constraints on selective codons.

GC3 compositional constraint effect				
	CGT	AGG	CTG	CTA
Pearson correlation (r)	−0.08583	0.14005	0.79525	−0.62947
Regression coefficient (r2)	0.0073	0.019613	0.63242	0.39623
*p* value	NS	NS	<0.0001	<0.0001
Slope	−0.002	0.007	0.0553	−0.113
Intercept	0.721	0.765	−0.729	1.037
AT3 compositional constraint effect				
Pearson correlation (r)	0.0858	−0.1400	−0.7952	0.6294
Regression coefficient (r2)	0.0073	0.0196	0.6324	0.3962
*p* value	NS	NS	<0.0001	<0.0001
Slope	0.0023	-0.007	-0.553	0.0113
Intercept	0.490	1.553	4.801	−0.1003

NS- Non-significant ***-p<0.0001.

### 3.10 Selectional Force is Dominant as Per Neutrality Analysis

**FIGURE 5:**
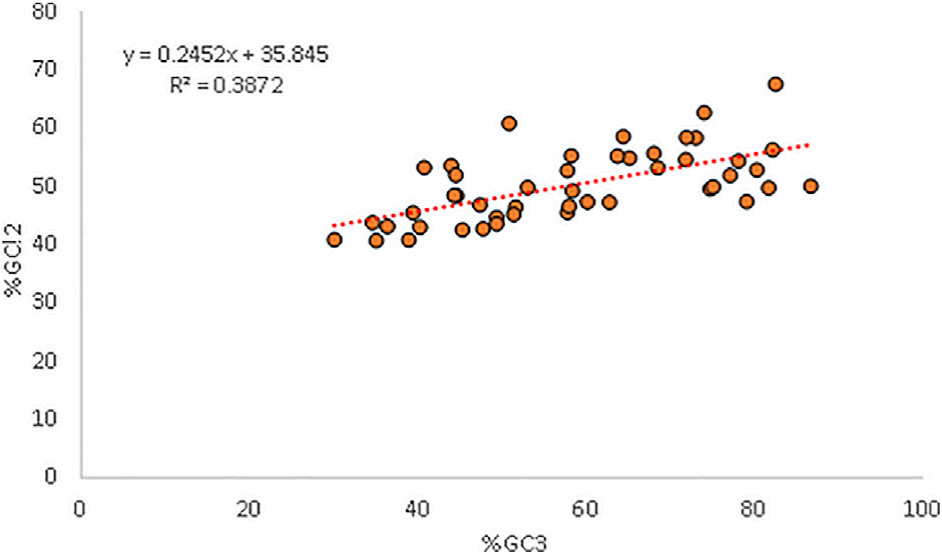
Regression plot analysis between GC3 and GC12 exhibiting the mutational and selection forces.

Percent GC12 and %GC3 had a positive correlation (r = 0.622, *p* < 0.001). A neutrality plot is drawn to precisely determine which force is the major force affecting codon usage and quantify it. The neutrality plot indicated that the relative neutrality is 24.52%, while the relative constraint was 75.48% for GC3 (Kumar et al., 2021). The GC12 content was affected by mutation pressure and natural selection with a ratio of = 24.52/75.48 = 0.324 (Figure 5).

### 3.11 Parity Analysis Refers to the Preference of Pyrimidines Over Purine at the Third Codon Position

The mean value of AT bias [A3/(A3 + T3)] was 0.498 ± 0.071 and GC bias [G3/(G3 + C3)] was 0.442 ± 0.076. The plot indicated that T and C are preferred over A and G (Zhang et al., 2018). When the overall nucleotide skew was observed, a positive value was obtained for both the AT and GC skews. Furthermore, the positive skew value indicated nucleotide A dominance over T and G over C. Cumulatively, the parity and skew analysis results indicate that overall A and G nucleotides are dominant, while T and C nucleotides are dominant at the third codon position.

### 3.12 Effect of Mutational Force of Composition Reveals Variable Mutational Force on Each of the Nucleotides

**FIGURE 6:**
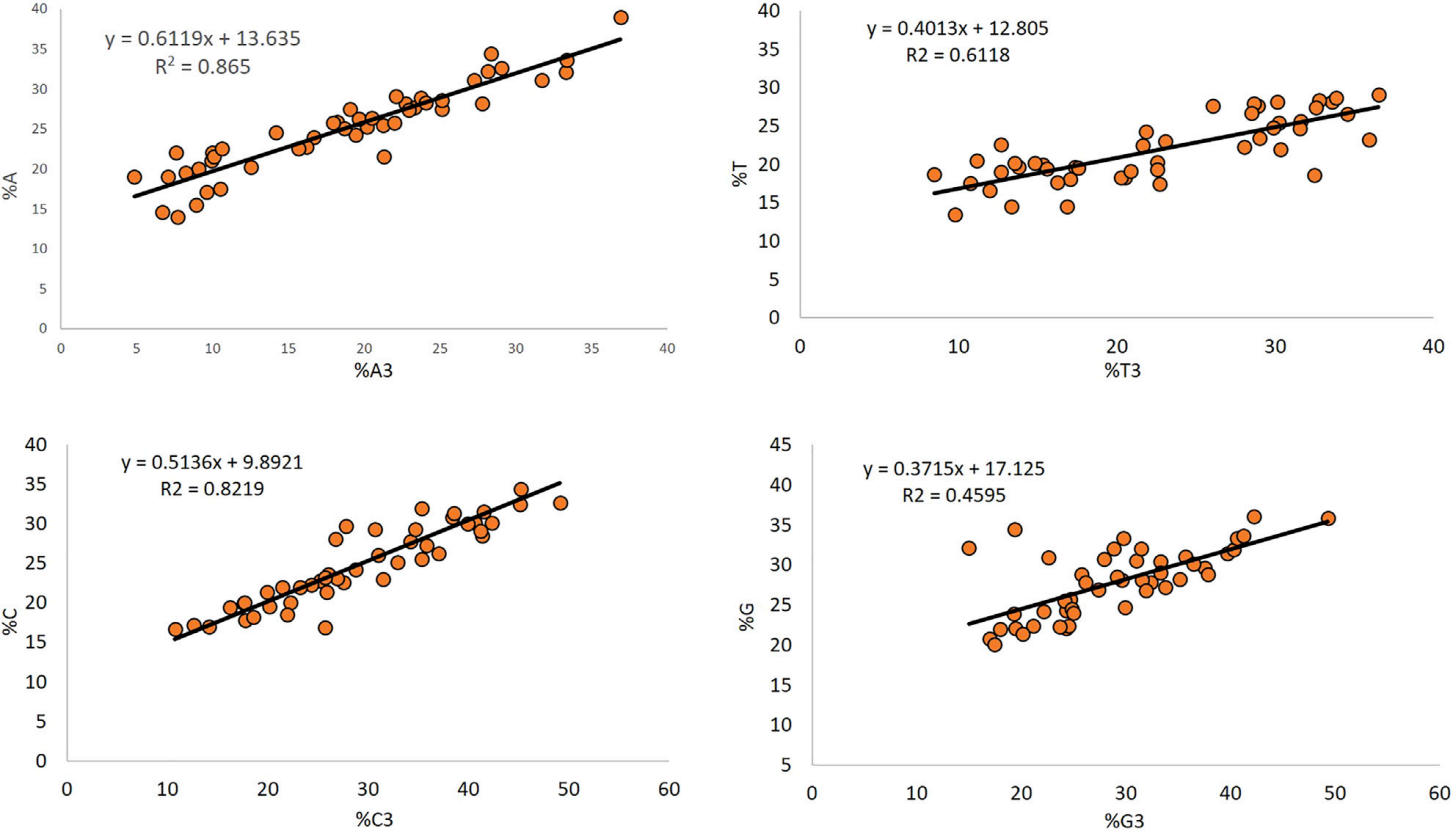
Determination of the effect of mutational force on composition by regression analysis.

The regression analysis between the overall composition and composition at the third position of codon indicates the mutational pressure (Uddin and Chakraborty, 2019), and it affected nucleotide A the maximum (67.19%), while it affected nucleotide G the least (37.15%). Nucleotides T and C contributed 40.13 and 51.36% to mutation, respectively (Figure 6).

### 3.13 Correspondence Analysis Indicated the Influence of Selectional Forces

Correspondence analysis on RSCU values of genes involved in dementia revealed a scattered distribution of genes, showing variability in codon usage. Axis 1 contributed for 41.58% and axis 2 contributed for 6.66% variation. Axis 1 contributed the maximum for variation, and both the GC- and AT-ending genes were located near axis 1, which indicates the effect of both the AT-ending and GC-ending codons on CUB (Lu, 2005). The genes had a major distribution along the first axis, suggesting other factors like selectional force on the codon usage of genes related to dementia (Wei et al., 2014). A biplot analysis revealed that codons AGA and CTG along the first axis and AGG and CGC along the second axis influenced CUB.

### 3.14 Gene Expression Level is Affected by Nucleotide Disproportion and Other Factors

CAI is a measure of the codon expression level of any gene, and higher CAI values reveal a higher expression level. CAI value was the highest for the *CTSD* gene (CAI = 0.849) and the least for the *VPS13A* gene (CAI = 0.673). A correlation analysis was done between the AT, GC, purine, pyrimidine, keto, and amino skews, and CAI to determine the possible connection between the gene expression level and nucleotide disproportion. The analysis revealed that except AT and GC skews, the gene expression level is negatively influenced by purine (r = −0.777, *p* < 0.001), pyrimidine (r = −0.805, *p* < 0.001), amino (r = −0.735, *p* < 0.001), and keto (r = −0.754, *p* < 0.001) skews. There was no correlation between the CAI and SCS, indicative of no effect of expression levels of genes on CUB. With the length of the protein, the CAI had a negative association (r = −0.303, *p* < 0.05), and it decreased with the increasing length of the protein. The protein expressivity in the genes associated with dementia is positively influenced by GC3 (r = 0.851, *p* < 0.001), while negative influenced by %GC12 (r = −0.303, *p* < 0.05). The GC3 content can be considered an indicator representing the extent of bias in nucleotide composition (Sablok et al., 2011). The association of GC3 with CAI indicated the effects of nucleotide bias on gene expression. CAI had a statistically significant (*p* < 0.05) negative association with AT-ending codons (all codons except CGA, CGT, TGT), while a significant positive association (*p* < 0.05) with GC-ending codons except codon AGG.

### 3.15 Translational Selection Effect

A P2 value higher than 0.5 indicated a bias toward the translational selection (A U et al., 2019). In the present study, the P2 value was 1.01, indicating a strong translation efficacy toward selectional force.

## 4 Discussion

The nucleotide composition gene is a crucial determinant of many of the properties and CUB. The composition might affect a protein’s physical properties, like stability and various functions that could be ascribed by the secondary structure [36]. In the present study, we found the highest percentage of %C and the lowest percentage of %T, while %A and %C nucleotides were almost equal. A lower occurrence of C and G nucleotides has been observed by Franzo et al. (2021) (Franzo et al., 2021). The GC content at the three positions is documented to be variable, and Song et al. (2017) (Song et al., 2017) reported the order %GC3>%GC2>%GC1 in peramine-coding genes of Epichloë species; however, in a highly expressed gene, a higher GC2 content was reported. In the present case, the average percent analysis of composition revealed variability in the % GC composition according to the position of the codon, and %GC1 and %GC3 compositions were almost equal while %GC2 composition was least.

Protein properties like GRAVY and AROMA are linked to the nucleotide composition, and CUB indicated that codon variations affect protein properties (Huang et al., 2017). CUB was negatively associated with the aliphatic index in the spike protein gene of infectious bronchitis virus (Makhija and Kumar, 2015), while positively associated with GRAVY and AROMO in genes of Ginkgo biloba (He et al., 2016). Protein indices like GRAVY and AROMA are the indices of natural selection (K handia et al., 2019). In the 47 genes associated with dementia, out of nine envisaged protein indices, only one physical property, viz. the frequency of neutral amino acid, was found linked with CUB, depicting that the effect of physical properties on CUB is at a low level in our study. Various nucleotide skews, including AT skew (A-T/A + T), GC skew (G-C/G + C), purine skew (A-G/A + G), pyrimidine skew (T-C/T + C), amino skew (A-C/A + C), and keto skew (T-G/T + G), explain nucleotide composition disproportion and nucleotide skew impacts on CUB. Correlation analysis between CUB and various skews revealed an association between the skews and CUB (Chakraborty et al., 2019). A positive association of CUB (SCS) with purine, pyrimidine, amino, and keto skew was observed in the present study. So, it can be inferred that codon bias will also increase with the increasing disproportion in nucleotide composition.

Codon usage bias correlates with GC composition and, generally, a very low or very high GC composition refers to a greater codon usage bias (Wan et al., 2004). In the present study, nucleotide A has a positive association with CUB at all the three positions of codons, and the GC content had a negative relationship with CUB at all three positions of the codon. Other researchers also found a relationship between codon bias and nucleotide composition suggestive of mutational force acting on codon bias (Deb et al., 2020). CpC dinucleotide abundance has been reported with the fine-tuning of gene expression also (Khandia et al., 2022). The positive association of odds ratio of A/T-containing dinucleotides (ApA, ApC, ApT, TpA, and TpT) and the positive association of odds ratio of G/C-containing dinucleotides (CpG, GpC, and GpG) with CUB indicated the presence of selectional forces in shaping codon usage.

Under-representation of CpG and TpA, dinucleotides are common in eukaryotes, and for CpG in mammalian genomes, the occurrence is at one-fifth of their expected frequency is supported by experimental pieces of evidence (Simmen, 2008). Our study also found under-represented CpG and TpA dinucleotides, apparently resulting from selectional forces (Munjal et al., 2020). The under-representation of CpG can be understood because in the CpG context, cytosine is found methylated in eukaryotes and becomes 5-methylcytosine. Methylated cytosine undergoes rapid deamination to give rise to thymidine. In further rounds of replication, in the case of endogenous mismatch repair enzymes failing in repairing this, CpG dinucleotide changes into TpG, or CpA (if the mutation occurred on the opposite strand) (Salser, 1978). TpA under-representation is present throughout the eukaryotic genomes (Karlin and Mrázek, 1997) and is attributed to the fact that TpA in the mRNA sequence (UpA) is prone to be a target by cellular RNases. Also, under-representation could be described based on its presence in two of three stop codons (Kumar et al., 2021). If we try to link the CpG deficit to the TpG excess, we found either over-representation or random representation of TpG dinucleotide in the genes evaluated in the present study. Further, if the CpG deficit is solely attributed to TpG conversion, there must be a negative correlation between CpG and TpG contents, but no such relation was observed in the present study. So, it further questions the theory of CpG methylation behind the CpG deficit.

Even though we did not find a correlation between the CpG and TpG/CpA, TpG dinucleotide-containing codons CTG and GTG were over-represented in 74.46 and 68.08% genes, respectively, while all CpG-containing codons (CGT, GCG, ACG, CCG, and TCG) were under-represented (in 53.19, 72.34, 57.44, 70.21, and 78.72% genes respectively). CTG codon has been over-represented in genes common in primary immunodeficiencies and cancer (Khandia et al., 2021). The same has been depicted in genes associated with brain iron accumulation (Alqahtani et al., 2021) (Alqahtani et al., 2021). With that, A/T-ending codons positively influenced CUB and G/C-ending codons negatively influenced CUB coupled with the fact that AT-ending codons were preferred, while GC-ending codons were not preferred. It indicated that with the usage of highly preferred codons, CUB is also increased. Biplot analysis revealed that AGA and CTG along the first axis and AGG and CGC along the second axis influenced CUB the most. In the study of Yang et al. (2010), other codons, including CGC (Arg), AGC (Ser), and GGC (Gly), were also found to be critical in influencing codon usage bias.

Codon usage bias correlates with GC composition, and with increasing GC composition, GC ending codon preference also increases (Wan et al., 2004). However, codon TTG behaves differently, and when GC ending codons positively influence gene expression, TTG showed an inverse relationship. Also, at one end, where all the GC ending codons show a positive relation with GC-ending codons, TTG showed a positive relationship with AT-ending codons. In the studies of Alqahtani et al. (2021), TTG has been shown to have an inverse relationship with the GC content, the gene set associated with neurodegeneration with iron accumulation. A codon preference affects the expression level of individual genes and the gene translation level of other proteins present in cells (Frumkin et al., 2018). This behavior cannot be explained based on compositional bias. The result follows the works of Kliman and Bernal (2005), who found in a high expression dataset of human genes, an exaggerated T→C transition rates are attributed to a higher number of CTG codons at the expense of TTG codons. Also, a decline in TTG numbers is observed in highly expressed genes suggestive of operative selection force.

Percent GC3 composition indicates CUB (Shen et al., 2015b) and base composition constraint (Das and Roy, 2021b). Regression analysis was done for unusual behavior displaying codons CGT and AGG, whether their CUB is driven by the compositional constraint. Moreover, CTG and CTA, the most over-represented and under-represented codons in the set of 47 genes, regressed with %GC3 as a reference. On the one hand, where 63.2 and 39.6% variations in the RSCU of CTG and CTA could be explained based on %GC3, CGT and AGG codons could explain only 0.74 and 1.14% variations in %GC3 indicative of a very poor association with compositional constraint.

CAI is a measure of the gene expression level, and highly expressed genes have CAI values near 1. Also, values close to 1 indicate that codons with highly RSCU values are used in the gene (K handia et al., 2019). The CAI value is independent of protein length and depends on amino acid frequency (Xia, 2007). However, in the present study, we found a negative association of CAI with the length of the protein, referring to the selection force favoring shorter proteins over larger proteins. Opposite results were obtained by Huang et al. (2017), who reported significant positive correlations of CAI with gene length in Taenia multiceps. GC3 was found to positively influence CAI, indicating the effect of nucleotide composition on gene expression (Sablok et al., 2011). CAI was negatively associated with AT-ending codons, while positively associated with highly preferred GC-ending codons.

Gene expression level is influenced by nucleotide disproportion also. Kleerebezem et al., 2003 suggested the effect of skewness in CUB. A negative GC skew value refers to the richness of C nucleotides over G, and likewise, a negative AT skew refers to the richness of T over A nucleotides (Nath Choudhury et al., 2017). In the cp genes, Zhang et al. (2013) reported more frequent usage of pyrimidines than purine at the third position of codons. In the present study, CAI was found to have no association with AT or GC skews but a significant (*p* < 0.05) negative correlation with a purine (A-G/A + G), pyrimidine (T-C/T + C), amino (A-C/A + C), and keto skews (T-G/T + G) suggests an effect of compositional disproportion on the gene expression level. A significant correlation of protein indices GRAVY and AROMO with the codon compositions at the third codon positions (A3s, T3s, G3s, C3s, and GC3s) showed natural selection as the principal force in shape codon usage in the evolution of canine distemper virus (Wang et al., 2020). In our study, all the relationships between nucleotide composition and protein indices were found (positive, negative, and no correlations), indicating the imperative role of selectional forces.

From the studies of various researchers, it is evident that CUB in any organism is the result of selectional and mutational forces and compositional constraints. Mutational force is the key force in shaping CUB in the *Gallus* genome (Rao et al., 2011), while compositional constraints are imperative in hemagglutinin gene in the H1N1 subtype of influenza A virus (Deka and Chakraborty, 2014). Sometimes, a combination of forces is involved (Chen et al., 2014; Trotta, 2016; Yengkhom et al., 2019; Khandia et al., 2021). Other forces, including mutational drift (Bulmer, 1991) and tRNA availability (Rocha, 2004), also affect codon usage.

A neutrality plot is generated to determine the decisive force between mutation and selection in CUB. When a correlation between GC12 and GC3 is present, it indicates mutational force likely working on all the codon positions (Jenkins and Holmes, 2003). In our study, a positive relationship between GC12 and GC3 indicated the presence of mutational pressure. Our results are in concordance with Uddin (2020), who also reported the same in genes associated with anxiety in humans. The regression plot indicated a strong selectional force where relative neutrality (mutational force) was 24.52%, while the relative constraint (natural selection) was 75.48%. The dominance of T and C over A and G in the parity plot again highlighted the role of selectional forces. The translational selection value greater than 0.5 in the present study also signified the importance of selectional pressure.

The regression analysis between the overall composition and composition at the third position of the codon is indicative of the role of mutational pressure acting in shaping the nucleotide composition [56]. In the present study, mutational forces affected the nucleotide A composition the maximum (67.19%). In comparison, it affected nucleotide G least (37.15%), inferring the effects of mutational force in determining the composition of a gene. A greater contribution of nucleotides G and A (68.48 and 71.19%, respectively) has been observed in the Anelloviridae genomes (Deb et al., 2021).

## 5 Conclusion

Overall, the analysis indicated the presence of mutational and selectional forces and compositional constraints in shaping codon usage. Nucleotide compositions greatly affected the bias and nucleotide A positively affected, and GC compositions negatively affected CUB at all codon positions. Mutational force affected nucleotide A the maximum and overall contributed 67.19% to the CUB. Based on the RSCU analysis, it is clear that GC-ending codons are over-represented and positively influence the CUB. Highly biased composition and over-representation of GC-ending codons were associated with higher expression levels. Based on the neutrality plot, parity plot, under-representation of TpA and CpG, and over the presentation of TpG dinucleotide, the preference of GC-ending codons over AT-ending codons, the high value of P2 and the relationship of gene expression with gene length, and unusual behavior of TTG codon, all point toward the dominant role of selectional force over mutational force.

## Data Availability

The datasets presented in this study can be found in online repositories. The names of the repository/repositories and accession number(s) can be found below: https://www.ncbi.nlm.nih.gov/gtr/.
