# Corrigendum: Codon usage is influenced by compositional constraints in genes associated with dementia

**DOI:** 10.3389/fgene.2025.1556255

**Published:** 2025-01-27

**Authors:** Taha Alqahtani, Rekha Khandia, Nidhi Puranik, Ali M. Alqahtani, Yahia Alghazwani, Saad Ali Alshehri, Kumarappan Chidambaram, Mohammad Amjad Kamal

**Affiliations:** ^1^ Department of Pharmacology, College of Pharmacy, King Khalid University, Abha, Saudi Arabia; ^2^ Department of Biochemistry and Genetics, Barkatullah University, Bhopal, India; ^3^ Department of Pharmacognosy, College of Pharmacy, King Khalid University, Abha, Saudi Arabia; ^4^ Institutes for Systems Genetics, Frontiers Science Center for Disease-Related Molecular Network, West China Hospital, Sichuan University, Chengdu, China; ^5^ King Fahd Medical Research Center, King Abdulaziz University, Jeddah, Saudi Arabia; ^6^ Department of Pharmacy, Faculty of Allied Health Sciences, Daffodil International University, Dhaka, Bangladesh; ^7^ Enzymoics, Novel Global Community Educational Foundation, Hebersham, NSW, Australia

**Keywords:** dementia, GC composition, compositional constraint, codon usage, nucleotide skew

In the published article, there was an error in the **Author** list, and authors Yahia Alghazwani and Saad Ali Alshehri were erroneously excluded. The corrected author list appears below:

“Taha Alqahtani, Rekha Khandia, Nidhi Puranik, Ali M Alqahtani, Yahia Alghazwani, Saad Ali Alshehri, Kumarappan Chidambaram and Mohammad Amjad Kamal.”

The authors apologize for this error and state that this does not change the scientific conclusions of the article in any way. The original article has been updated.

